# Crystal structure and Hirshfeld surface analysis of 2-(4-nitro­phen­yl)-2-oxoethyl picolinate

**DOI:** 10.1107/S2056989019014105

**Published:** 2019-10-29

**Authors:** T. N. Sanjeeva Murthy, C. S. Chidan Kumar, S. Naveen, M. K. Veeraiah, Kakarla Raghava Reddy, Ismail Warad

**Affiliations:** a Department of Chemistry, Sri Siddhartha Academy of Higher Education, Tumkur 572 107, Karnataka, India; b Department of Chemistry, Vidya Vikas Institute of Engineering & Technology, Visvesvaraya Technological University, Alanahally, Mysuru 570 028, India; c Department of Physics, School of Engineering and Technology, Jain University, Bangalore 562 112, India; d Department of Chemistry, Sri Siddhartha Institute of Technology, Tumkur 572 105, Karnataka, India; e School of Chemical & Biomolecular Engineering, The University of Sydney, Sydney, NSW, Australia; f Department of Chemistry, Science College, An-Najah National University, PO Box 7, Nablus, West Bank, Palestinian Territories

**Keywords:** crystal structure, R_{2}^{2}(10) ring motif, inter­moleculsr inter­actions, Hirshfeld surface analysis

## Abstract

2-(4-Nitro­phen­yl)-2-oxoethyl picolinate, C_14_H_10_N_2_O_5_, was synthesized under mild conditions. The chemical and mol­ecular structures were confirmed by single-crystal X-ray diffraction analysis. The mol­ecules are linked by inversion into centrosymmetric dimers *via* weak inter­molecular C—H⋯O inter­actions, forming *R*_2_^2^(10) ring motifs, and further strengthened by weak π–π inter­actions. Hirshfeld surface analyses, the *d*_norm_ surfaces, electrostatic potential and two-dimensional fingerprint (FP) plots were used to verify the contributions of the different inter­molecular inter­actions within the supra­molecular structure. The shape-index surface shows that two sides of the mol­ecules are involved with the same contacts in neighbouring mol­ecules and curvedness plots show flat surface patches that are characteristic of planar stacking.

## Abstract 2

2-(4-Nitro­phen­yl)-2-oxoethyl picolinate was synthesized under mild conditions. The chemical and mol­ecular structure was confirmed by single-crystal X-ray diffraction studies. The mol­ecules are related by inversion into centrosymmetric dimers *via* weak C—H⋯O inter­molecular inter­actions, and further strengthened by weak π–π inter­actions. A qu­anti­fication of the inter­molecular contacts in the crystal were estimated using Hirshfeld surface analysis and two-dimensional fingerprint plots.

## Chemical context   

Derivatives of phenacyl bromide have found significant application in the identification of organic acids (Rather & Reid, 1919 ▸). In organic chemistry, phenacyl benzoate is a derivative of an acid, formed by reaction between an acid and phenacyl bromide. The syntheses of phenacyl esters have many advantages in organic chemistry because they are usually solids and provide a useful means of characterizing acids and phenols. Phenacyl esters are useful for the photoremoval of protecting groups for carb­oxy­lic acids in organic synthesis and biochemistry. These com­pounds can be photolysed under neutral and mild conditions (Sheehan *et al.*, 1973 ▸; Ruzicka *et al.*, 2002 ▸; Literák *et al.*, 2006 ▸). They also find application in the field of synthetic chemistry, such as in the synthesis of oxazoles and imidazoles (Huang *et al.*, 1996 ▸), as well as with benzoxazepine (Gandhi *et al.*, 1995 ▸). In continuation of our work on the synthesis of these ester derivaties (Kumar *et al.*, 2014 ▸), we report herein the crystal and mol­ecular structures of 2-(4-nitro­phen­yl)-2-oxoethyl picolinate.
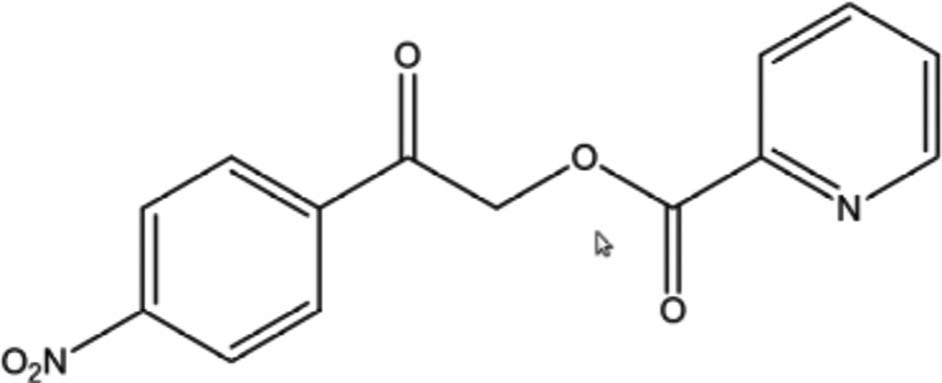


## Structural commentary   

**Figure 1:**
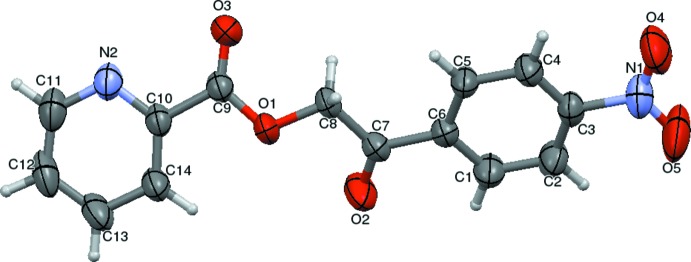
The mol­ecular structure of the title com­pound, indicating the atom-numbering scheme and with displacement ellipsoids drawn at the 50% probability level.

The mol­ecular structure of the title com­pound is shown in Fig. 1 ▸, and bond lengths and angles are listed in Table 1 ▸. The com­pound is com­posed of two aromatic rings (4-­nitro­phenyl and pyridine) linked by C—C(=O)—O—C(=O) bonds forming a bridge. The unique mol­ecular conformation of this com­pound is characterized by three torsion angles, *viz.* τ_1_ (N2—C10—C9—O3), τ_2_ (C7—C8—O1—C9) and τ_3_ (O2—C7—C6—C1), whereby τ_1_ [−6.1 (2)°] signifies the apparent co­planarity of the mean planes of the pyridine and adjacent carbonyl rings at the connecting bridge. The torsion angle value of τ_2_ = −147.02 (11)° between the two carbonyl groups indicates a –*anti­clinal* conformation. Likewise, owing to a substitution on the functional group, the title com­pound experiences steric repulsion between the substituent and adjacent carbonyl groups, which can influence the torsion angle [τ_3_ = 2.4 (2)%] and resulting in a +*synclinal* conformation. The bond lengths and angles are normal and the mol­ecular conformation is characterized by a dihedral angle of 31.58 (8)° between the mean planes of the two aromatic rings. The nitro group lies nearly in the plane of the phenyl ring, as indicated by the torsion angle values of −4.7 (2) and −5.1 (2)° for C4—C3—N1—O4 and C2—C3—N1—O5, respectively.

## Supra­molecular features   

**Figure 2:**
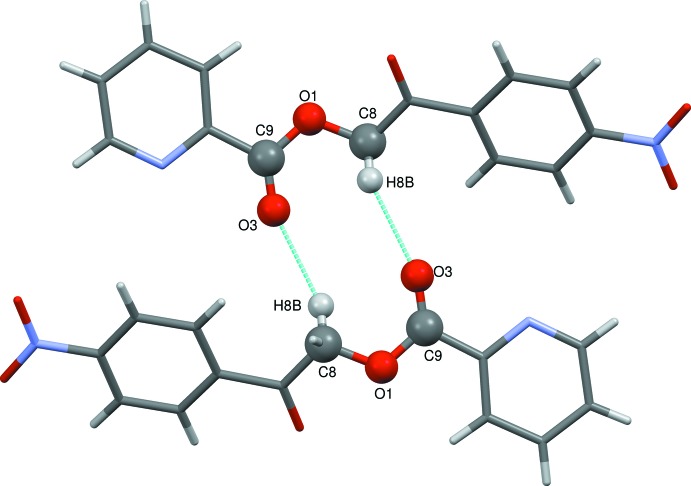
A view of two mol­ecules of the title com­pound linked by inversion into centrosymmetric dimers by weak C8—H8*B*⋯O3 inter­molecular inter­actions forming an 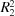
(10) ring motif. **[See Note 1]**

**Figure 3:**
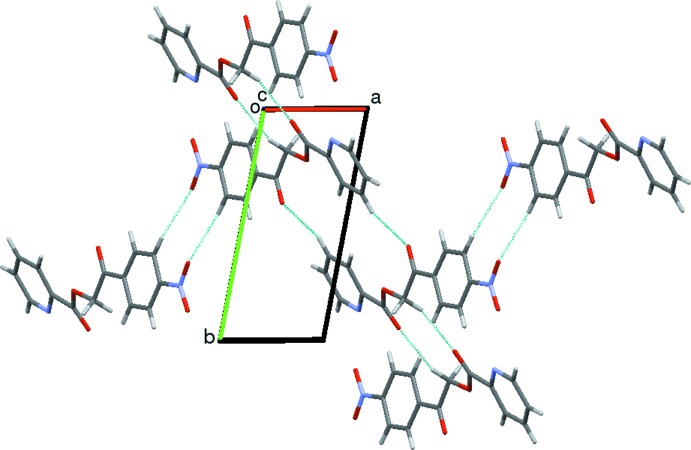
The packing of mol­ecules of the title com­pound in the *ab* plane, viewed along the *c* axis. Cyan dashed lines indicate weak inter­molecular C—H⋯O inter­actions forming 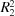
(10) ring motifs.

There are no classical hydrogen bonds in the structure. However, the structure is consolidated by weak C—H⋯O inter­molecular inter­actions. Specifically, singular weak inter­molecular C8—H8*B*⋯O3(−*x*, −*y*, −*z*) inter­actions stabilize the supra­molecular architecture by forming 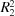
(10) ring motifs and chains along [011] (Fig. 2 ▸). The mol­ecular structure is also stabilized by weak inter­molecular C—O⋯*Cg*, N—O⋯*Cg* and *Cg*⋯*Cg* inter­actions. The hydrogen-bond geometry and lone pair-π inter­actions are listed in Table 2 ▸. The mol­ecule also exhibits *Cg*⋯*Cg* inter­actions, *i.e.* *Cg*1⋯*Cg*1 [*Cg*1 is the centroid of the N2/C10/C14–C11 ring; *Cg*⋯*Cg* distance = 4.6293 (10) Å, α = 0°, β = 42.1°, the perpendicular distance of *Cg*1 on itself = 3.4332 (7) Å (symmetry code: *x* − 1, *y*, *z*)] and *Cg*2⋯*Cg*2 [*Cg*2 is the centroid of the pyridine ring;; *Cg*⋯*Cg* distance = 4.6292 (10) Å, α = 0°, β = 40.3°, γ = 40.3° and the perpendicular distance of *Cg*2 on itself = 3.5322 (6) Å (symmetry code: *x* + 1, *y*, *z*)]. These weak inter­molecular inter­actions link the mol­ecules to form a one-dimensional chain along the *c* axis and the mol­ecules exhibit layered stacking (Fig. 3 ▸).

## Hirshfeld surface analysis   

**Figure 4:**
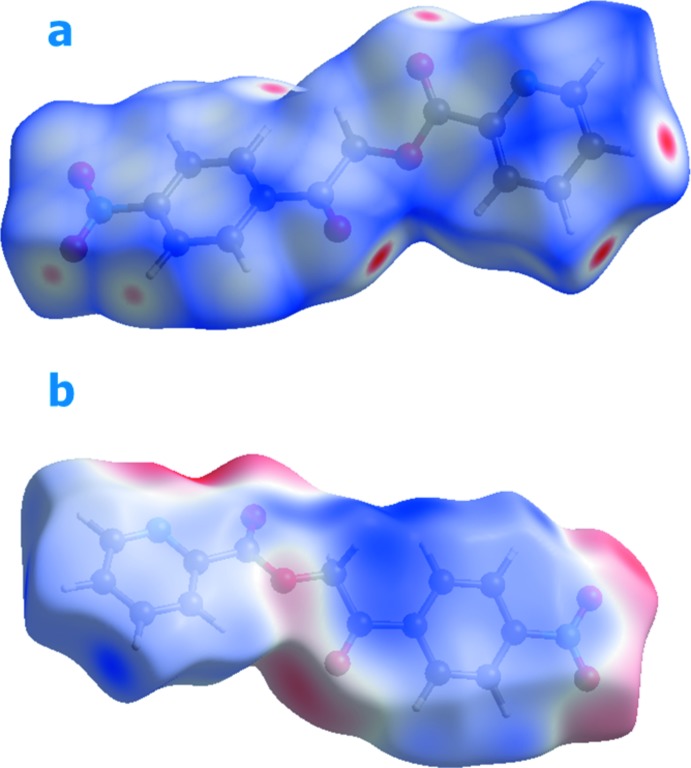
A view of the three-dimensional Hirshfeld surface of the title com­pound mapped over *d*_norm_.

Hirshfeld surfaces and fingerprint plots (McKinnon *et al.*, 2007 ▸) were generated for the title com­pound based on the crystallographic information file (CIF) using *CrystalExplorer* (Wolff *et al.*, 2012 ▸). Hirshfeld surfaces enable the visualization of inter­molecular inter­actions by different colours and colour intensity, representing short or long contacts and indicating the relative strengths of the inter­actions. Figs. 4 ▸ and 5 ▸ show the Hirshfeld surfaces mapped over *d*_norm_ (−0.196 to 1.128 a.u.) and shape-index (−1.0 to 1.0 a.u.), respectively. The calculated volume inside the Hirshfeld surface is 311.97 Å^3^ in the area of 305.78 Å^3^.

In Fig. 4 ▸, the dark spots near the C and O atoms result from C—H⋯O inter­actions, which play a significant role in the mol­ecular packing of the title com­pound. The Hirshfeld surfaces illustrated in Fig. 4 ▸ also reflect the involvement of different atoms in the inter­molecular inter­actions through the appearance of blue and red regions around the participating atoms, which correspond to positive and negative electrostatic potential, respectively. The shape-index surface clearly shows that the two sides of the mol­ecules are involved in the same contacts with neighbouring mol­ecules and the curvedness plots show flat surface patches characteristic of planar stacking.

**Figure 6:**
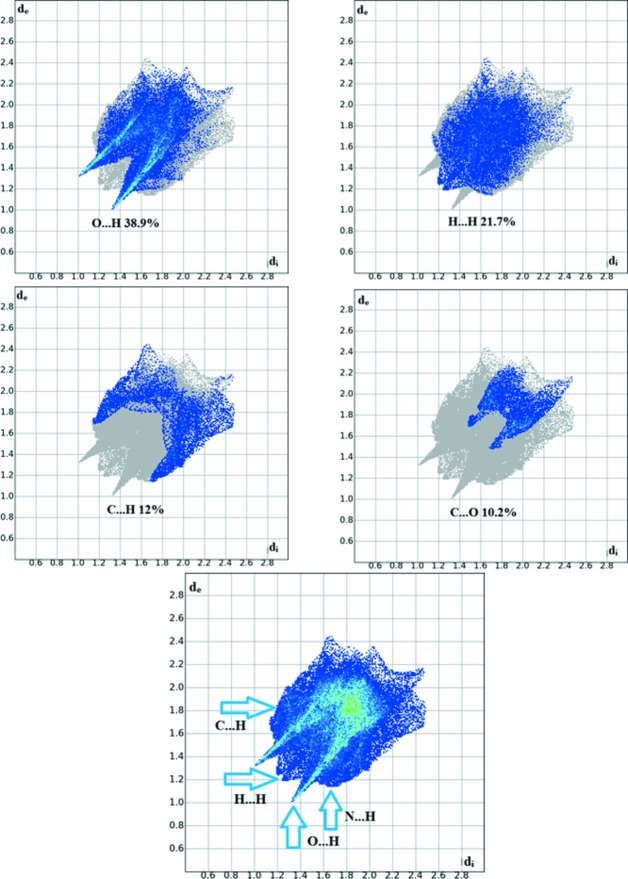
Two-dimensional fingerprint plots of the title com­pound, showing the percentage contributions of all inter­actions, and the individual types of inter­actions.

The overall two-dimensional fingerprint plot for the title com­pound and those delineated into O⋯H/H⋯H, H⋯H, C⋯H/H⋯C, C⋯O/O⋯C and N⋯H/H⋯N contacts are illustrated in Fig. 6 ▸; the percentage contributions from the different inter­atomic contacts to the Hirshfeld surfaces are as follows: O—H 38.9%, H—H 21.7%, C—H12%, C—O 10.2% and N—H 8.2%, as shown in the two-dimensional fingerprint plots, respectively, in Fig. 6 ▸. The percentage contributions for the other inter­molecular contacts are less than 5% in the Hirshfeld surface mapping.

## Database survey   

**Figure 7:**
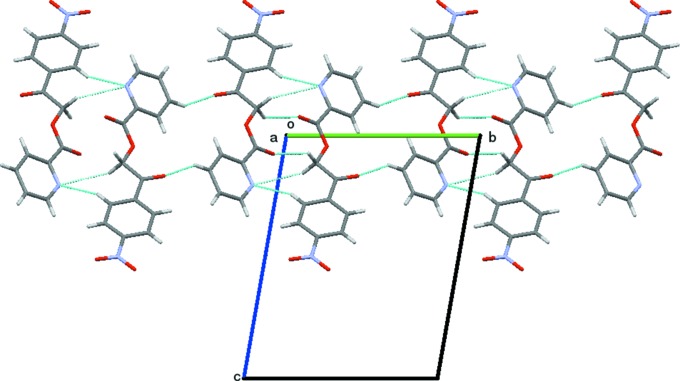
Packing of the molecules when viewed down along the *a* axis. The dashed lines represent C—H⋯O hydrogen bonds.

A search of the Cambridge Structural Database (CSD, Version 5.40, last update May 2019; Groom *et al.*, 2016 ▸) using 2-oxo-2-phenyl­ethyl benzoate as the main skeleton revealed the presence of a number structures containing a moiety similar to the title com­pound, but with different substituents on the terminal phenyl rings. These include the following: 2-oxo-2-phenyl­ethyl benzoate, 2-(4-bromo­phen­yl)-2-oxoethyl 4-meth­oxy­benzoate, 2-(4-bromo­phen­yl)-2-oxoethyl 4-chloro­benzoate, 2-(4-bromo­phen­yl)-2-oxoethyl 4-bromo­benzoate, 2-(4-chloro­phen­yl)-2-oxoethyl 2-meth­oxy­benzoate, 2-(4-bromo­phen­yl)-2-oxoethyl 2-meth­oxy­benzoate, 2-(4-chloro­phen­yl)-2-oxoethyl 2,4-di­fluoro-benzoate, 2-(4-chloro­phen­yl)-2-oxoethyl 2,4-di­fluoro­benzoate, 2-(4-chloro­phen­yl)-2-oxoethyl benzoate, 2-(4-chloro­phen­yl)-2-oxoethyl 4-hy­droxy­benzoate, 2-(4-bromo­phen­yl)-2-oxoethyl 2-methyl­benzoate, 2-(4-chloro­phen­yl)-2-oxoethyl 4-methyl­benzoate, 2-(4-bromo­phen­yl)-2-oxoethyl 4-hy­droxy­benzoate, 2-(4-bromo­phen­yl)-2-oxoethyl 4-methyl­benzoate, 2-(2,4-di­chloro­phen­yl)-2-oxoethyl 4-meth­oxy­benzoate, 2-(4-fluoro­phen­yl)-2-oxoethyl 4-meth­oxy­benzoate and 2-(4-chloro­phen­yl)-2-oxoethyl 3,4-di­meth­oxy­benzoate (Fun *et al.*, 2011*a* ▸,*b* ▸,*c* ▸,*d* ▸,*e* ▸,*f* ▸,*g* ▸,*h* ▸,*i* ▸,*j* ▸,*k* ▸,*l* ▸,*m* ▸,*n* ▸,*o* ▸), 2-(4-fluoro­phen­yl)-2-oxoethyl 2-meth­oxy­benzoate (Isloor *et al.*, 2012 ▸), 1-(4-bromo­phen­yl)-2-(2-chloro­phen­oxy)ethanone (Shenvi *et al.*, 2012 ▸) and 2,4-di­chloro­benzyl 2-meth­oxy­benzoate (Isloor *et al.*, 2013 ▸). In these 19 com­pounds, the dihedral angles between the phenyl rings are in the range 3.2 (2)–85.92 (10)°. The difference may arise from the weak inter­molecular inter­actions between adjacent mol­ecules (Fig. 7 ▸).

## Synthesis and crystallization   

The title com­pound was synthesized as per the procedure of Kumar *et al.* (2014 ▸). A mixture of 2-bromo-1-(4-nitro­phen­yl)ethanone (0.2 g, 0.5 mmol), potassium carbonate (0.087 g, 0.63 mmol) and nicotinic acid (0.079 g, 0.65 mmol) in di­methyl­formamide (5 ml) was stirred at room temperature for 5 h. After com­pletion of the reaction, the reaction mixture was poured into ice-cold water. The solid product obtained was filtered off, washed with water and recrystallized from ethanol [m.p. 407–410 K, determined with a Stuart Scientific (UK) apparatus].

## Refinement   

Crystal data, data collection and structure refinement details are summarized in Table 3 ▸. H atoms on C atoms were positioned geometrically (C—H = 0.95–0.99 Å) and refined using a riding model, with *U*_iso_(H) = 1.2 or 1.5*U*_eq_(C).

## Supplementary Material

Crystal structure: contains datablock(s) global, I. DOI: 10.1107/S2056989019014105/jj2216sup1.cif

Structure factors: contains datablock(s) I. DOI: 10.1107/S2056989019014105/jj2216Isup2.hkl

CCDC references: 1449658, 1449658

Additional supporting information:  crystallographic information; 3D view; checkCIF report

## Figures and Tables

**Figure 5:**
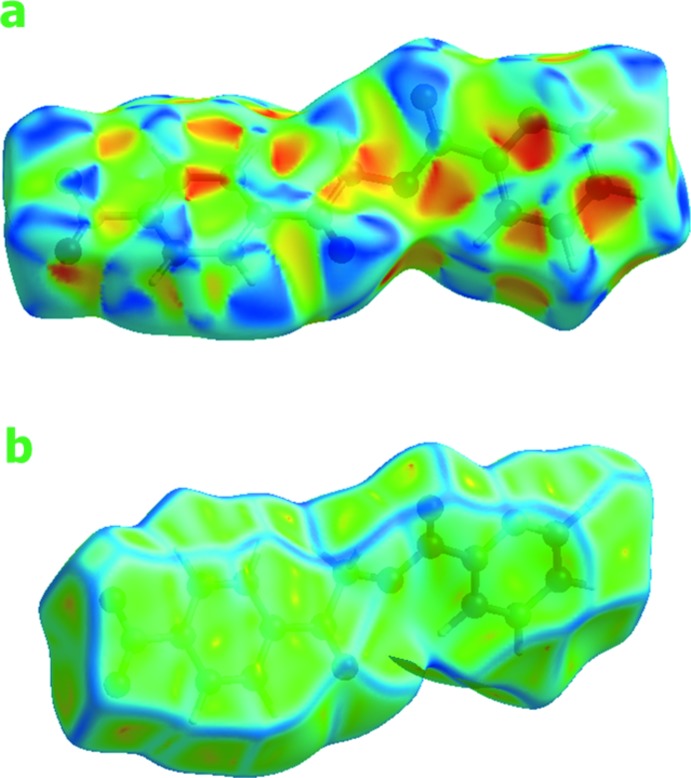
Hirshfeld surface of the title com­pound mapped with shape-index and curvedness.

**Table 1:** Selected geometric parameters (Å, °)

O1—C8	1.4329 (17)	O5—N1	1.211 (2)
O1—C9	1.3374 (16)	N1—C3	1.4761 (19)
O2—C7	1.2021 (18)	N2—C10	1.3372 (18)
O3—C9	1.1969 (17)	N2—C11	1.339 (2)
O4—N1	1.205 (2)		
			
C8—O1—C9	116.39 (10)	O2—C7—C8	121.71 (13)
O4—N1—O5	123.38 (16)	O1—C8—C7	108.11 (11)
O4—N1—C3	118.83 (14)	O1—C9—O3	123.96 (13)
O5—N1—C3	117.79 (15)	O1—C9—C10	111.08 (11)
C10—N2—C11	115.93 (13)	O3—C9—C10	124.96 (12)
N1—C3—C2	118.29 (14)	N2—C10—C9	114.56 (12)
N1—C3—C4	118.83 (13)	N2—C10—C14	124.07 (13)
O2—C7—C6	120.57 (13)	N2—C11—C12	123.99 (16)

**Table 2:** Hydrogen-bond geometry (Å, °) *Cg*1 and *Cg*2 are the centroids of the pyridine and nitrophenyl rings, respectively.

*D*—H⋯*A*	*D*—H	H⋯*A*	*D*⋯*A*	*D*—H⋯*A*
C5—H5*A*⋯O3^i^	0.93	2.55	3.2283 (18)	130
C8—H8*B*⋯O3^i^	0.97	2.45	3.2681 (17)	141
C12—H12*A*⋯O5^ii^	0.93	2.52	3.396 (3)	157
C13—H13*A*⋯O2^iii^	0.93	2.47	3.277 (2)	146
C9—O3⋯*Cg*1		3.35 (1)	3.4735 (16)	86 (1)
C7—O2⋯*Cg*2		3.58 (1)	3.8788 (15)	67 (1)
N1—O4⋯*Cg*2		3.76 (1)	3.5479 (16)	71 (1)
N1—O5⋯*Cg*2		3.68 (1)	3.5479 (16)	74 (1)

Symmetry codes: (i) 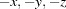; (ii) 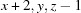; (iii) 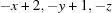.

**Table 3:** Experimental details

Crystal data	
Chemical formula	C_14_H_10_N_2_O_5_
*M*_r_	286.24
Crystal system, space group	Triclinic, *P* 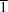
Temperature (K)	297
*a*, *b*, *c* (Å)	4.6292 (4), 10.6563 (9), 13.3592 (11)
α, β, γ (°)	99.136 (1), 93.426 (1), 100.556 (1)
*V* (Å^3^)	636.95 (9)
*Z*	2
Radiation type	Mo *K*α
μ (mm^−1^)	0.12
Crystal size (mm)	0.41 × 0.27 × 0.16

Data collection	
Diffractometer	Bruker APEXII DUO CCD area-detector
Absorption correction	Multi-scan (*SADABS*; Bruker, 2012 ▸)
*T*_min_, *T*_max_	0.953, 0.981
No. of measured, independent and observed [*I* > 2σ(*I*)] reflections	21701, 3496, 2571
*R*_int_	0.022
(sin θ/λ)_max_ (Å^−1^)	0.690

Refinement	
*R*[*F* ^2^ > 2σ(*F* ^2^)], *wR*(*F* ^2^), *S*	0.046, 0.136, 1.07
No. of reflections	3496
No. of parameters	190
H-atom treatment	H-atom parameters constrained
Δρ_max_, Δρ_min_ (e Å^−3^)	0.24, −0.19

Computer programs: *APEX2* (Bruker, 2012 ▸), *SAINT* (Bruker, 2012 ▸), *SHELXS97* (Sheldrick, 2008 ▸), *SHELXL97* (Sheldrick, 2008 ▸), *Mercury* (Macrae *et al.*, 2008 ▸), *SHELXL2015* (Sheldrick, 2015 ▸) and *PLATON* (Spek, 2009 ▸).

## supplementary crystallographic information

### Crystal data

**Table:**

C_14_H_10_N_2_O_5_	*Z* = 2
*M**_r_* = 286.24	*F*(000) = 296
Triclinic, *P*1	*D*_x_ = 1.492 Mg m^−^^3^
Hall symbol: -P 1	Mo *K*α radiation, λ = 0.71073 Å
*a* = 4.6292 (4) Å	Cell parameters from 2571 reflections
*b* = 10.6563 (9) Å	θ = 1.6–29.4°
*c* = 13.3592 (11) Å	µ = 0.12 mm^−^^1^
α = 99.136 (1)°	*T* = 297 K
β = 93.426 (1)°	Rectangle, white
γ = 100.556 (1)°	0.41 × 0.27 × 0.16 mm
*V* = 636.95 (9) Å^3^	

### Data collection

**Table:**

Bruker APEXII DUO CCD area-detector diffractometer	3496 independent reflections
Radiation source: fine-focus sealed tube	2571 reflections with *I* > 2σ(*I*)
Graphite monochromator	*R*_int_ = 0.022
Detector resolution: 18.4 pixels mm^-1^	θ_max_ = 29.4°, θ_min_ = 1.6°
φ and ω scans	*h* = −6→6
Absorption correction: multi-scan (SADABS; Bruker, 2012)	*k* = −14→14
*T*_min_ = 0.953, *T*_max_ = 0.981	*l* = −18→18
21701 measured reflections	

### Refinement

**Table:**

Refinement on *F*^2^	Primary atom site location: structure-invariant direct methods
Least-squares matrix: full	Secondary atom site location: difference Fourier map
*R*[*F*^2^ > 2σ(*F*^2^)] = 0.046	Hydrogen site location: inferred from neighbouring sites
*wR*(*F*^2^) = 0.136	H-atom parameters constrained
*S* = 1.07	*w* = 1/[σ^2^(*F*_o_^2^) + (0.0588*P*)^2^ + 0.1188*P*] where *P* = (*F*_o_^2^ + 2*F*_c_^2^)/3
3496 reflections	(Δ/σ)_max_ < 0.001
190 parameters	Δρ_max_ = 0.24 e Å^−^^3^
0 restraints	Δρ_min_ = −0.18 e Å^−^^3^

### Special details

**Table:**

Geometry. Bond distances, angles etc. have been calculated using the rounded fractional coordinates. All su's are estimated from the variances of the (full) variance-covariance matrix. The cell esds are taken into account in the estimation of distances, angles and torsion angles
Refinement. Refinement on F^2^ for ALL reflections except those flagged by the user for potential systematic errors. Weighted R-factors wR and all goodnesses of fit S are based on F^2^, conventional R-factors R are based on F, with F set to zero for negative F^2^. The observed criterion of F^2^ > 2sigma(F^2^) is used only for calculating -R-factor-obs etc. and is not relevant to the choice of reflections for refinement. R-factors based on F^2^ are statistically about twice as large as those based on F, and R-factors based on ALL data will be even larger.

### Fractional atomic coordinates and isotropic or equivalent isotropic displacement parameters (Å^2^)

**Table:**

	*x*	*y*	*z*	*U*_iso_*/*U*_eq_	
O1	0.5089 (2)	0.21852 (9)	0.04455 (7)	0.0528 (3)	
O2	0.3481 (3)	0.40340 (11)	0.16679 (10)	0.0824 (5)	
O3	0.2932 (2)	0.05168 (10)	−0.07629 (8)	0.0578 (3)	
O4	−0.6315 (3)	0.13249 (15)	0.50999 (11)	0.0961 (6)	
O5	−0.5484 (4)	0.33778 (16)	0.55148 (12)	0.1149 (7)	
N1	−0.5128 (3)	0.23923 (15)	0.49895 (10)	0.0653 (5)	
N2	0.7107 (3)	0.12934 (12)	−0.20395 (9)	0.0563 (4)	
C1	−0.0180 (4)	0.38578 (14)	0.32225 (12)	0.0581 (5)	
C2	−0.2018 (4)	0.37345 (15)	0.39938 (12)	0.0614 (5)	
C3	−0.3152 (3)	0.25177 (14)	0.41686 (10)	0.0493 (4)	
C4	−0.2565 (3)	0.14168 (14)	0.36073 (11)	0.0547 (4)	
C5	−0.0737 (3)	0.15511 (13)	0.28293 (11)	0.0512 (4)	
C6	0.0465 (3)	0.27660 (12)	0.26370 (9)	0.0426 (3)	
C7	0.2433 (3)	0.29607 (13)	0.17993 (10)	0.0466 (4)	
C8	0.2962 (3)	0.17779 (13)	0.11235 (10)	0.0491 (4)	
C9	0.4795 (3)	0.14691 (12)	−0.04857 (10)	0.0435 (4)	
C10	0.7087 (3)	0.20214 (12)	−0.11282 (10)	0.0442 (4)	
C11	0.9099 (4)	0.17721 (17)	−0.26372 (13)	0.0659 (6)	
C12	1.1060 (4)	0.29307 (18)	−0.23613 (14)	0.0707 (6)	
C13	1.1009 (4)	0.36515 (16)	−0.14264 (14)	0.0675 (5)	
C14	0.8961 (3)	0.31902 (13)	−0.07891 (12)	0.0536 (4)	
H1A	0.06280	0.46770	0.30960	0.0700*	
H2A	−0.24750	0.44630	0.43860	0.0740*	
H4A	−0.33680	0.06020	0.37440	0.0660*	
H5A	−0.03160	0.08170	0.24340	0.0610*	
H8A	0.36960	0.12060	0.15300	0.0590*	
H8B	0.11330	0.13120	0.07380	0.0590*	
H11A	0.91590	0.12910	−0.32780	0.0790*	
H12A	1.24020	0.32180	−0.28060	0.0850*	
H13A	1.23200	0.44370	−0.12200	0.0810*	
H14A	0.88590	0.36610	−0.01480	0.0640*	

### Atomic displacement parameters (Å^2^)

**Table:**

	*U*^11^	*U*^22^	*U*^33^	*U*^12^	*U*^13^	*U*^23^
O1	0.0520 (5)	0.0536 (5)	0.0472 (5)	−0.0055 (4)	0.0231 (4)	0.0038 (4)
O2	0.1022 (9)	0.0475 (6)	0.0962 (9)	−0.0035 (6)	0.0567 (8)	0.0123 (6)
O3	0.0560 (6)	0.0544 (6)	0.0562 (6)	−0.0052 (4)	0.0168 (4)	0.0034 (4)
O4	0.1105 (11)	0.0952 (10)	0.0910 (10)	0.0128 (8)	0.0615 (9)	0.0306 (8)
O5	0.1705 (16)	0.0994 (11)	0.0930 (10)	0.0483 (11)	0.0884 (11)	0.0174 (9)
N1	0.0719 (8)	0.0818 (10)	0.0506 (7)	0.0243 (7)	0.0280 (6)	0.0176 (7)
N2	0.0651 (7)	0.0588 (7)	0.0479 (6)	0.0142 (6)	0.0207 (5)	0.0096 (5)
C1	0.0717 (9)	0.0420 (7)	0.0613 (9)	0.0079 (6)	0.0242 (7)	0.0080 (6)
C2	0.0757 (10)	0.0527 (8)	0.0587 (9)	0.0193 (7)	0.0263 (7)	0.0035 (7)
C3	0.0501 (7)	0.0608 (8)	0.0394 (6)	0.0129 (6)	0.0150 (5)	0.0098 (6)
C4	0.0638 (8)	0.0489 (7)	0.0510 (7)	0.0032 (6)	0.0225 (6)	0.0102 (6)
C5	0.0617 (8)	0.0416 (7)	0.0489 (7)	0.0045 (6)	0.0223 (6)	0.0035 (5)
C6	0.0424 (6)	0.0427 (6)	0.0412 (6)	0.0037 (5)	0.0103 (5)	0.0057 (5)
C7	0.0451 (6)	0.0453 (7)	0.0479 (7)	0.0010 (5)	0.0143 (5)	0.0089 (5)
C8	0.0500 (7)	0.0483 (7)	0.0478 (7)	0.0002 (5)	0.0222 (5)	0.0087 (5)
C9	0.0437 (6)	0.0432 (6)	0.0456 (7)	0.0082 (5)	0.0143 (5)	0.0102 (5)
C10	0.0461 (6)	0.0452 (6)	0.0455 (7)	0.0116 (5)	0.0175 (5)	0.0126 (5)
C11	0.0812 (11)	0.0742 (10)	0.0521 (8)	0.0272 (9)	0.0313 (8)	0.0170 (7)
C12	0.0771 (10)	0.0764 (11)	0.0763 (11)	0.0270 (9)	0.0479 (9)	0.0368 (9)
C13	0.0670 (9)	0.0548 (8)	0.0852 (11)	0.0043 (7)	0.0367 (8)	0.0236 (8)
C14	0.0573 (8)	0.0468 (7)	0.0581 (8)	0.0057 (6)	0.0256 (6)	0.0107 (6)

### Geometric parameters (Å, º)

**Table:**

O1—C8	1.4329 (17)	C7—C8	1.4973 (19)
O1—C9	1.3374 (16)	C9—C10	1.4998 (19)
O2—C7	1.2021 (18)	C10—C14	1.3737 (19)
O3—C9	1.1969 (17)	C11—C12	1.375 (3)
O4—N1	1.205 (2)	C12—C13	1.362 (3)
O5—N1	1.211 (2)	C13—C14	1.387 (2)
N1—C3	1.4761 (19)	C1—H1A	0.9300
N2—C10	1.3372 (18)	C2—H2A	0.9300
N2—C11	1.339 (2)	C4—H4A	0.9300
C1—C2	1.382 (2)	C5—H5A	0.9300
C1—C6	1.386 (2)	C8—H8A	0.9700
C2—C3	1.369 (2)	C8—H8B	0.9700
C3—C4	1.369 (2)	C11—H11A	0.9300
C4—C5	1.387 (2)	C12—H12A	0.9300
C5—C6	1.3822 (19)	C13—H13A	0.9300
C6—C7	1.5006 (19)	C14—H14A	0.9300
			
C8—O1—C9	116.39 (10)	N2—C11—C12	123.99 (16)
O4—N1—O5	123.38 (16)	C11—C12—C13	119.00 (17)
O4—N1—C3	118.83 (14)	C12—C13—C14	118.62 (16)
O5—N1—C3	117.79 (15)	C10—C14—C13	118.39 (14)
C10—N2—C11	115.93 (13)	C2—C1—H1A	120.00
C2—C1—C6	120.27 (14)	C6—C1—H1A	120.00
C1—C2—C3	118.56 (14)	C1—C2—H2A	121.00
N1—C3—C2	118.29 (14)	C3—C2—H2A	121.00
N1—C3—C4	118.83 (13)	C3—C4—H4A	121.00
C2—C3—C4	122.88 (14)	C5—C4—H4A	121.00
C3—C4—C5	118.09 (13)	C4—C5—H5A	120.00
C4—C5—C6	120.56 (13)	C6—C5—H5A	120.00
C1—C6—C5	119.64 (13)	O1—C8—H8A	110.00
C1—C6—C7	117.81 (12)	O1—C8—H8B	110.00
C5—C6—C7	122.54 (12)	C7—C8—H8A	110.00
O2—C7—C6	120.57 (13)	C7—C8—H8B	110.00
O2—C7—C8	121.71 (13)	H8A—C8—H8B	108.00
C6—C7—C8	117.70 (12)	N2—C11—H11A	118.00
O1—C8—C7	108.11 (11)	C12—C11—H11A	118.00
O1—C9—O3	123.96 (13)	C11—C12—H12A	120.00
O1—C9—C10	111.08 (11)	C13—C12—H12A	121.00
O3—C9—C10	124.96 (12)	C12—C13—H13A	121.00
N2—C10—C9	114.56 (12)	C14—C13—H13A	121.00
N2—C10—C14	124.07 (13)	C10—C14—H14A	121.00
C9—C10—C14	121.36 (12)	C13—C14—H14A	121.00
			
C9—O1—C8—C7	−147.02 (11)	C4—C5—C6—C1	−0.5 (2)
C8—O1—C9—O3	−1.63 (19)	C4—C5—C6—C7	−179.49 (13)
C8—O1—C9—C10	178.12 (11)	C1—C6—C7—O2	2.4 (2)
O4—N1—C3—C2	174.45 (15)	C1—C6—C7—C8	−175.97 (13)
O4—N1—C3—C4	−4.7 (2)	C5—C6—C7—O2	−178.60 (14)
O5—N1—C3—C2	−5.1 (2)	C5—C6—C7—C8	3.1 (2)
O5—N1—C3—C4	175.83 (15)	O2—C7—C8—O1	6.80 (19)
C11—N2—C10—C9	178.95 (13)	C6—C7—C8—O1	−174.90 (11)
C11—N2—C10—C14	−0.4 (2)	O1—C9—C10—N2	174.17 (12)
C10—N2—C11—C12	0.4 (3)	O1—C9—C10—C14	−6.46 (18)
C6—C1—C2—C3	0.6 (3)	O3—C9—C10—N2	−6.1 (2)
C2—C1—C6—C5	−0.1 (2)	O3—C9—C10—C14	173.28 (14)
C2—C1—C6—C7	178.99 (15)	N2—C10—C14—C13	0.0 (2)
C1—C2—C3—N1	−179.64 (15)	C9—C10—C14—C13	−179.32 (14)
C1—C2—C3—C4	−0.6 (2)	N2—C11—C12—C13	−0.1 (3)
N1—C3—C4—C5	179.10 (13)	C11—C12—C13—C14	−0.4 (3)
C2—C3—C4—C5	0.0 (2)	C12—C13—C14—C10	0.4 (2)
C3—C4—C5—C6	0.5 (2)		

### Hydrogen-bond geometry (Å, º)

**Table:**

*D*—H···*A*	*D*—H	H···*A*	*D*···*A*	*D*—H···*A*
C5—H5*A*···O3^i^	0.93	2.55	3.2283 (18)	130
C8—H8*B*···O3^i^	0.97	2.45	3.2681 (17)	141
C12—H12*A*···O5^ii^	0.93	2.52	3.396 (3)	157
C13—H13*A*···O2^iii^	0.93	2.47	3.277 (2)	146
C9—O3···*Cg*1		3.35 (1)	3.4735 (16)	86 (1)
C7—O2···*Cg*2		3.58 (1)	3.8788 (15)	67 (1)
N1—O4···*Cg*2		3.76 (1)	3.5479 (16)	71 (1)
N1—O5···*Cg*2		3.68 (1)	3.5479 (16)	74 (1)

Symmetry codes: (i) −*x*, −*y*, −*z*; (ii) *x*+2, *y*, *z*−1; (iii) −*x*+2, −*y*+1, −*z*.
